# Reintervention of Residual Aortic Dissection after Type A Aortic Repair: Results of a Prospective Follow-Up at 5 Years

**DOI:** 10.3390/jcm12062363

**Published:** 2023-03-18

**Authors:** Alizée Porto, Virgile Omnes, Michel A. Bartoli, Ron Azogui, Noémie Resseguier, Mariangela De Masi, Laurence Bal, Laura Imbert, Nicolas Jaussaud, Pierre Morera, Alexis Jacquier, Pierre-Antoine Barral, Vlad Gariboldi, Marine Gaudry

**Affiliations:** 1 Department of Cardiac Surgery, Timone Hospital, Assistance Publique-Hôpitaux de Marseille, 13005 Marseille, France; 2 Department of Vascular Surgery, Timone Aortic Center, Timone Hospital, Assistance Publique-Hôpitaux de Marseille, 13005 Marseille, France; 3 Department of Epidemiology and Public Health Cost, Assistance Publique-Hôpitaux de Marseille, 13005 Marseille, France; 4 Department of Radiology, Timone Hospital, Assistance Publique-Hôpitaux de Marseille, 13005 Marseille, France

**Keywords:** residual aortic dissection, hybrid repair, branched aortic arch endograft, long-term results, type A repair, reintervention

## Abstract

Background After a type A aortic dissection repair, a patent false lumen in the descending aorta is the most common situation encountered, and is a well-known risk factor for aortic growth, reinterventions and mortality. The aim of this study was to analyze the long-term results of residual aortic dissection (RAD) at a high-volume aortic center with prospective follow-up. Methods In this prospective single-center study, all patients operated for type A aortic dissection between January 2017 and December 2022 were included. Patients without postoperative computed tomography scans or during follow-up at our center, and patients without RAD were excluded. The primary endpoint was all-cause mortality during follow-up for patients with RAD. The secondary endpoints were perioperative mortality, rate of distal aneurysmal evolution, location of distal aneurysmal evolution, rate of distal reinterventions, outcomes of distal reinterventions, and aortic-related death during follow-up. Results In total, 200 survivors of RAD comprised the study group. After a mean follow-up of 27.2 months (1–66), eight patients (4.0%) died and 107 (53.5%) had an aneurysmal progression. The rate of distal reintervention was 19.5% (39/200), for malperfusion syndrome in seven cases (3.5%) and aneurysmal evolution in 32 cases (16.0%). Most reinterventions occurred during the first 2 years (82.1%). Twenty-seven patients were treated for an aneurysmal evolution of RAD including aortic arch with hybrid repair in 21 cases and branched aortic arch endoprosthesis in six cases. In the hybrid repair group, there was no death, and the rate of morbidity was 28.6% (6/21) (one minor stroke, one pulmonary complication, one recurrent paralysis with complete recovery and three major bleeding events). In the branched endograft group, there was no death, no stroke, and no paraplegia. There was one case (16.7%) of carotid dissection. Complete aortic remodeling or complete FL thrombosis on the thoracic aorta was found in 18 cases (85.7%) and in five cases (83.3%) in the hybrid and branched endograft groups, respectively. Conclusions: Despite a critical course in most cases of RAD, with a high rate of aneurysmal evolution and reintervention, the long-term mortality rate remains low with a close follow-up and a multidisciplinary management in an expert center.

## 1. Introduction

Acute type A aortic dissection is an uncommon disease with an incidence of 3.0 per 100,000/year [1]. However, it is a life-threatening disease with a mortality rate that increases 1–2% per hour within the initial 48 h [2,3]. Open repair with ascending aortic replacement is the gold standard, and it improves survival with mortality at 30 days ranging between 10% and 20% in expert centers [4,5].

Among the survivors, a patent false lumen (FL) in the descending aorta is the most common situation encountered, ranging from 43% to 77.5% of cases, and is a well-known risk factor for aortic growth and mortality [6,7,8,9,10,11]. Close follow-up of residual aortic dissection (RAD) is essential to improve the long-term survival of these complex patients [5]. The rate of reinterventions on the descending thoracic aorta after type A aortic dissection repair is estimated to be between 10 and 40%. These reinterventions intend to prevent late rupture of the dissected aorta but remain challenging cases since RAD involves the aortic arch. Indeed, currently in aortic centers around the world, hemiarch replacement at the acute phase is the most common repair [12], with long-term survival in favor of proximal repair versus extensive repair [13].

Several surgical techniques have been proposed for redo surgery after type A repair: total open aortic arch repair, hybrid repair and total endovascular aortic arch repair with branched and fenestrated endoprostheses associated with distal thoracic endovascular aortic repair (TEVAR). Total open aortic arch or frozen elephant trunk repair in cases of redo surgery seems to be associated with a high rate of morbi-mortality, especially regarding neurological risk [14,15]. Hybrid repair for RAD is safe and associated with good anatomical results [16].

In the last decade, endovascular aortic arch repair has emerged as a less invasive alternative for these complex pathologies with a low morbi-mortality rate in this indication [17,18].

There are no recommendations for the management of these patients, so they require discussion with a multidisciplinary meeting and case-by-case decision making.

Several risk factors are known to promote aneurysmal evolution of the descending aorta after type A aortic repair: young age, male sex, aortic valve bicuspid, connective tissue diseases, aortic diameter > 40 mm, patent FL, absence of resection of the primary entry tear or new entry tear and the ratio between the true lumen (TL) and false lumen TL/FL < 1 and evolution of the false lumen volume at 3 months [7,9,19,20]. All these factors may be used to identify patients who may benefit from early endovascular intervention to improve their long-term survival [6,19].

The aim of this study was to analyze the long-term results of RAD after type A aortic repair at a high-volume aortic center with prospective follow-up.

## 2. Patients and Methods

The institutional review board approved the project (approval number 2019-48). The individual written informed consent was obtained for each patient.

### 2.1. Study Population

In this prospective single-center study, all patients operated for type A aortic dissection between January 2017 and December 2022 were included. Patients without postoperative computed tomography (CT) scans (not alive after intensive care unit), patients without CT scan follow-up at our center, and patients without RAD were excluded.

Demographic characteristics and preoperative and intraoperative variables were collected.

### 2.2. Endpoints

The primary endpoint was all-cause mortality during follow-up for patients with RAD.

The secondary endpoints were perioperative mortality, rate of distal aneurysmal evolution, location of distal aneurysmal evolution, rate of distal reinterventions, outcomes of distal reinterventions (indications, type of repair, morbi-mortality, long-term anatomical results), aortic-related death during follow-up, and risk factor of reintervention.

### 2.3. Follow-Up

All patients had an immediate postoperative CT scan and underwent clinical and radiological follow-up at 3, 6 and 12 months and annually in the case of favorable progress.

### 2.4. CT Protocol

All patients underwent postoperative and follow-up examinations with a three-phase CT scan.

Image analysis and measurements were performed using three-dimensional imaging software (OSIRIX software, Geneva, Switzerland). The maximal aortic diameter measurements were performed on the perpendicular axis according to the centerline using a semiautomated centerline algorithm at three different levels of the aorta (ascending aorta, aortic arch and descending thoracic aorta).

FL patency was assessed as FL enhancement anywhere in the downstream aorta on arterial or venous-phase CT. The FL was also considered patent if only partial thrombosis was observed, while disappearance of the FL was considered to indicate complete FL thrombosis.

Complete aortic remodeling was defined as complete aortic healing.

### 2.5. Surgical Procedures

#### 2.5.1. Initial Surgery for TAAD

Initial surgery for acute type A aortic dissection was performed in our center with cardiopulmonary bypass (CPB) and circulatory arrest (CA), retrograde cold blood cardioplegia, moderate hypothermia and anterograde cerebral perfusion when possible or deep hypothermia. Replacement of the ascending aorta, hemiarch aorta, partial arch replacement, or total arch replacement was performed depending on the location of the primary entry tear, as detailed in a previous study [21]. Aortic root replacement with a composite prosthesis according to the modified Bentall procedure or aortic root repair (Tirone David) was performed in patients with dilatation of the aortic root or an aortic root damaged by the entry tear. In our center, intervention of aortic valve resuspension with supracoronary hemiarch replacement remains the first-line therapy.

#### 2.5.2. Distal Reintervention on the Descending Thoracic aorta

The indications for RAD reintervention included complicated RAD (malperfusion syndrome, pain, rupture) or aneurysmal evolution (rapid aortic growth > 5 mm/6 months, aortic diameter > 55 mm). Validation of these interventions and the choice of surgical procedure for all cases were decided in a multidisciplinary meeting including cardiac surgeons, vascular surgeons, anesthesiologists, interventional radiologists, and cardiologists.

Endovascular therapy was our first-line therapy for descending thoracic aortic dissection.

The decision to extend the proximal landing zone was based on the location of the main residual entry tear (most of the time, at the distal anastomosis of the ascending aortic repair or in the aortic arch; more rarely, in the descending thoracic aorta). 

In the absence of RAD in the aortic arch and when the entry tear was in the descending thoracic aorta, we performed TEVAR on the descending thoracic aorta.

In cases of RAD in the aortic arch, we performed hybrid repair or branched aortic arch endoprosthesis.

### 2.6. Management of the Aortic Arch

#### 2.6.1. Hybrid Repair

Hybrid treatment with TEVAR and open supra-aortic debranching in at least two steps remains the first-line therapy at our center when the RAD involves the aortic arch, as previously described [16]. Complete supra-aortic debranching was performed by a prosthetic bypass between the ascending aortic replacement, innominate artery (IA), left common carotid artery (LCCA), and left subclavian artery (LSA) through a median sternotomy. In cases of previous IA debranching (during type A repair), we performed an intra-thoracic debranching without CPB with direct or indirect reimplantation of the LCCA in young patients without comorbidities or an extrathoracic debranching with a carotid-to-carotid prosthetic bypass in elderly patients with comorbidities.

#### 2.6.2. Branched Aortic Arch Endoprosthesis

Since 2020, in selected cases (contraindication of hybrid repair, absence of supra-aortic dissection), endovascular repair of the aortic arch with a branched endograft was performed.

All patients were treated with a branch endograft including one or two branches (relay branch, Terumo aortic, Miami, US and A-Branch, Cook Medical, Bloomington, IN, USA).

After systemic heparinization of 100 IU/kg, the device was advanced in the aortic arch and deployed under rapid pacing. We performed a surgical approach to the common carotids for branch delivery and femoral access for endoprosthesis delivery.

#### 2.6.3. TEVAR

Procedures were performed in a hybrid suite (Discovery IGS 730, GE Healthcare, Chicago, IL, USA). Transesophageal ultrasound was used systematically to ensure the correct positioning of the guide in the true lumen. Two different stent grafts were used: C-TAG (WL Gore & Associates Inc. Flagstaff, AZ, USA) and Terumo Aortic (Terumo aortic, Miami, FL, USA).

The distal extension of the stent graft was based on the distal extension of the dissected aortic aneurysm.

In addition to TEVAR, we have added a bare stent deployment in the thoraco-abdominal aorta (the Zenith dissection endovascular stent (ZDES), Cook Medical, Bloomington, IN, USA) to induce remodeling of the distal dissected aorta (stent-assisted balloon-induced intimal disruption and relamination in aortic dissection repair—STABILISE technique) [22,23]. This technique was chosen when anatomical criteria were favorable (maximum aortic diameter less than 42 mm, absence of aortic angulation, thrombus in the FL) during the first year following the type A repair [24]. In other cases, we performed TEVAR alone, KNICKERBOCKER [25] or Candyplug.

The diameter of the stent graft was sized based on the proximal and distal sealing zones with a maximum 20% oversizing compared with the native aorta or ascending aortic graft.

Cerebrospinal fluid drainage was performed when there was extensive coverage of the thoracic aorta (>250 mm). Spinal drains were placed preoperatively by anesthesiologists, a small (14-gauge) epidural catheter was placed using anatomic landmarks, with needle placement at L3–L4, and the catheter was advanced 10 cm after entering the dura. Antiplatelets were stopped 5 days earlier. Spinal fluid was drained to obtain a spinal fluid pressure < 10 mmHg before device deployment.

### 2.7. Statistical Analysis

Means and range or standard deviation were used to describe continuous variables; categorical variables were described as numbers and frequencies. For categorical variables, the relationship between variables was studied using the chi-square test or Fisher’s exact test, as appropriate. The Mann–Whitney U test was used for continuous variables. The normality of the distribution of variables was assessed with the Shapiro–Wilk test.

Overall survival was estimated using Kaplan–Meier method. Cumulative incidence of reintervention was estimated using the time-to-event approach and taking into account the occurrence of death before reintervention as a competing event. Fine and Gray models were built to estimate subdistribution hazard ratios with their 95% confidence intervals to quantify the association between patients’ characteristics and reintervention risk over time.

All statistical tests were two-sided, and for all analyses, a *p*-value of <0.05 was considered statistically significant. Statistical analyses were performed using IBM SPSS Statistics 20.0 (IBM, Inc., New York, NY, USA) and R software version 4.2.2 (R Foundation for statistical Computing, Vienna, Austria).

## 3. Results

In this study, 357 patients were treated for acute type A aortic dissection, the hospital mortality rate was 12.6% (45/357), 88 patients (24.6%) had no residual dissection, and 24 were lost to follow-up (6.7%). In total, 200 survivors of RAD comprised the study group.

The mean age was 62.2 +/−11.6 years, 72.5% (145) were male, and 6.0% (12/200) had Marfan and related syndromes.

Demographic data are presented in Table 1.

### 3.1. All-Cause Mortality of RAD

After a mean follow-up of 27.2 months (1–66), 8 patients (4.0%) died during follow-up: two from pancreatic cancer, two from COVID-19, one from influenza, one from unknown cause, one from cerebral tumor and one from aortic rupture (death after the first surgery pending endovascular exclusion).

The rate of aortic-related death was 0.5% (1/200).

**Figure 1:**
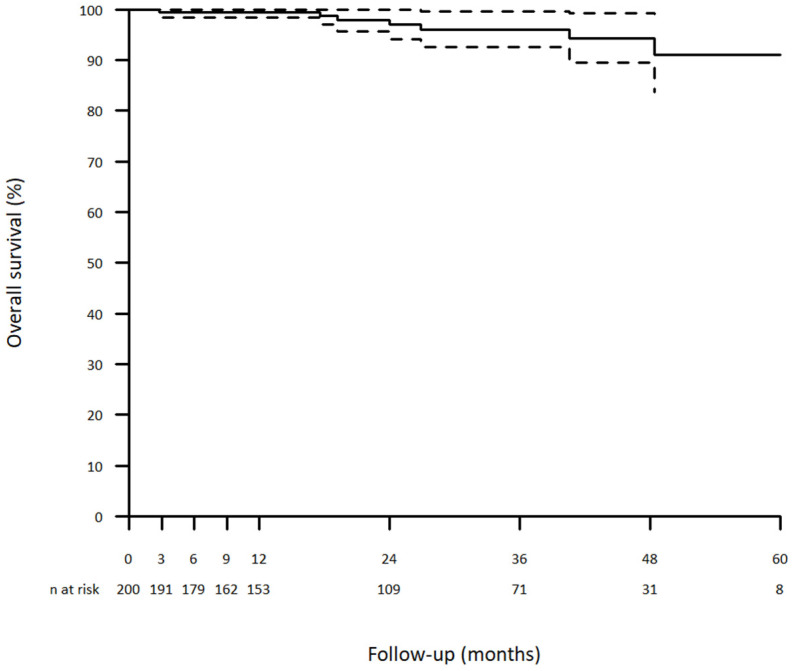
Overall survival curve (+: censored data).

The Kaplan–Meier estimated survival rates at 1, 2, 3 and 5 years were 99.5%, 97.1%, 96.1% and 91.1%, respectively (Figure 1).

### 3.2. Distal Aneurysm Evolution

**Figure 2:**
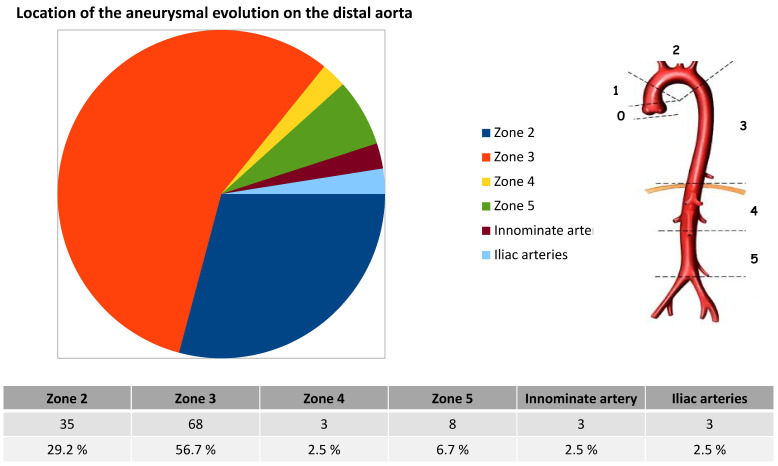
Location of the aneurysmal evolution on the distal aorta.

The rate of aneurysmal progression was 53.5% (107/200), and among the aneurysmal changes, 68 (34%) involved segment three, 35 (17.5%) involved segment two, 11 (5.5%) involved segments four and five, three (1.5%) involved the innominate artery (IA), and three (1.5%) involved the iliac arteries (Figure 2).

### 3.3. Distal Reintervention

**Figure 3:**
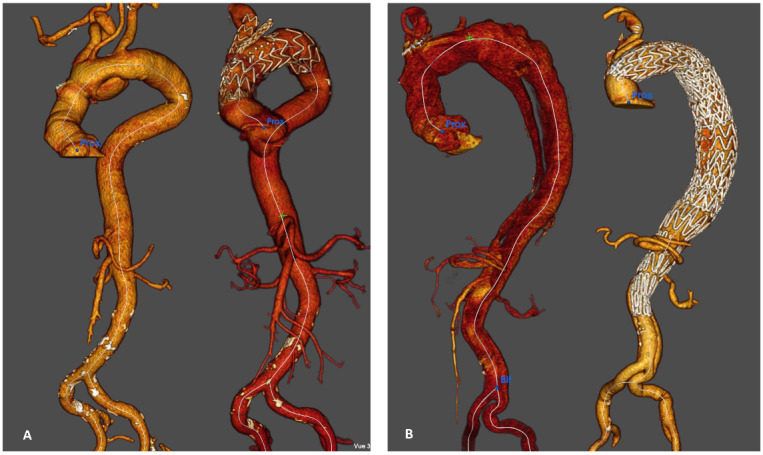
Branched endograft and hybrid repair for aneurysmal evolution of residual aortic dissection. (**A**): pre and post operative CT scan (volume rendering) after branched endograft for aneurysmal evolution limited to the aortic arch. (**B**): pre and post operative CT scan (volume rendering) after hybrid repair associated with TEVAR and STABILISE technique for a thoracic aneurysmal evolution.

The rate of distal reoperation was 19.5% (39/200): six branched aortic arch endoprostheses (Figure 3A), 21 hybrid treatments (Figure 3B), one conventional IA repair, four TEVARs alone, one open abdominal repair, one endovascular abdominal aortic repair, one fenestrated endovascular abdominal aortic repair, two iliac stenting and three renal stenting.

**Figure 4:**
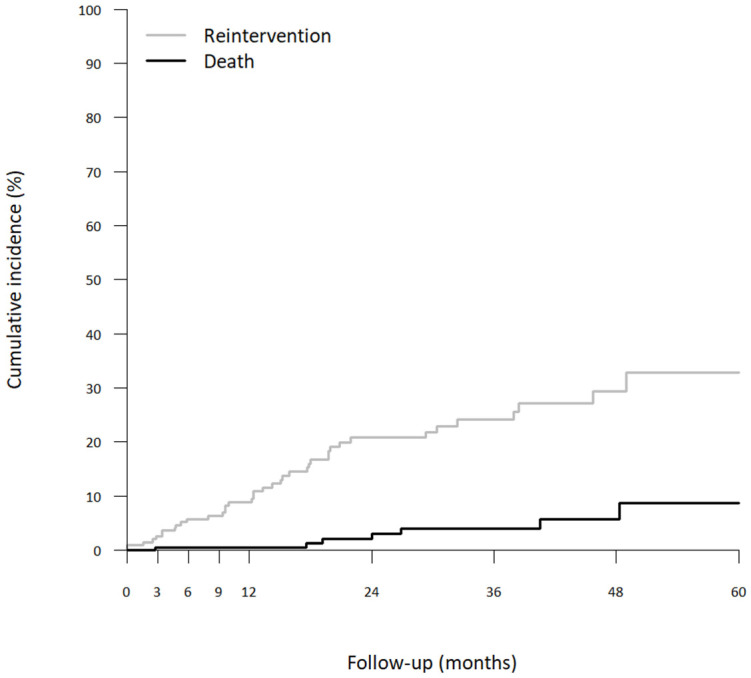
Cumulative incidence of reintervention using the time-to-event approach and considering the occurrence of death before reintervention as a competing event.

The cumulative incidence of reintervention at 1, 2, 3 and 5 years were 8.9%, 20.8%, 24.1% and 32.8%, respectively (Figure 4).

### 3.4. Indications

Indications for distal reintervention were malperfusion syndrome in seven cases (3.5%) and aneurysmal evolution in 32 cases (16.0%), among which 27 involved the aortic arch. No patient was treated for an aortic rupture. 

The demographic data and type of repair of these 27 patients are shown in Table 2.

During aneurysmal evolution, the mean maximum aortic diameter was 55 mm (43–76).

The mean delay between type A repair and reintervention was 15.3 months (1–48). Most reinterventions (32/39) occurred during the first 2 years (82.1%).

### 3.5. Perioperative Results of Distal Reintervention

#### 3.5.1. Results after Hybrid Repair

We performed extra-thoracic debranching with intercarotid prosthetic bypass (patients with previous IA debranching during type A repair) and LSA reimplantation in four patients and intra-thoracic debranching with complete supra-aortic debranching in 17 patients. Among these 17 patients, in four cases, the previous ascending aortic replacement was short, and a redo replacement of the ascending aorta with CPB and CA was necessary. In 14 patients, debranching was performed by a prosthetic bypass between the previous ascending aortic replacement, IA, left common carotid artery (LCCA), and left subclavian artery (LSA) with CPB.

There was no death, and the rate of morbidity was 28.6% (6/21): one minor stroke with complete recovery, one pulmonary complication, one recurrent paralysis with complete recovery and three major bleeding events, including one tamponade, one major renal bleeding needing renal embolization and one bleeding complication secondary to spinal drains (a medullar hematoma with cauda equina syndrome).

After a mean follow-up of 26.3 months (range 2–50), complete aortic remodeling or complete FL thrombosis on the thoracic aorta was found in 18 cases (85.7%), and partial FL thrombosis was found in three cases (14.3%) due to type IB endoleaks.

#### 3.5.2. Results after Branched Aortic Arch Endoprosthesis

One patient was treated with an endograft including one branch for the LCCA. The patient had previous IA debranching during type A repair and left subclavian artery transposition was necessary.

In five other cases, endografts included two branches, IA and LCCA in four cases associated with LSA debranching and LCCA and LSA in one case with previous IA debranching. In one of these patients, the device moved backward, and we performed a carotid-to-carotid prosthetic bypass with a single branch in the LCCA.

There was no death, no stroke, and no paraplegia. There was one case (16.7%) of carotid dissection.

After a mean follow-up of 13.5 months (range 1–26), complete aortic remodeling or complete FL thrombosis on the thoracic aorta was found in five cases (83.3%), and partial FL thrombosis was found in one case (16.7%) due to type IB endoleak.

We compared the results of hybrid repair and branched endograft for patients treated for aneurysmal evolution including aortic arch evolution, in Table 3.

#### 3.5.3. Results for Other Reinterventions

Among one conventional IA repair (with CPB and CA), four TEVARs alone, one open abdominal repair, one endovascular abdominal aortic repair, one fenestrated endovascular abdominal aortic repair, two iliac stenting and three renal stenting, there were no perioperative deaths or complications.

### 3.6. Risk Factors for Reinterventions

Marfan syndrome and bicuspid aortic valve were significantly associated with the risk of reintervention (HR = 3.19 CI_95_ = [1.34–7.56], *p* = 0.01 and HR = 4.55 CI_95_ = [2.36–8.76], *p* < 0.01 respectively). 

## 4. Discussion

In this study, with twice as many patients included as in our previous publication [21], we confirmed that RAD is a serious disease associated with a critical course for almost half of patients. This finding confirms that close follow-up in an expert center is essential. Indeed, operative survival does not guarantee freedom from subsequent aortic events, as most operative survivors have a persistent, dissected residual aorta, often with a patent FL [26]. In the international registry (IRAD), the survival rates of patients after type A aortic dissection treated with surgery were 96.1%+/−2.4% and 90.5%+/−3.9% at 1 and 3 years, respectively [2]. Yeh et al. reported a survival rate free from descending aortic aneurysm formation or descending aorta operation of 74.7% at 3 years [7]. The long-term mortality of these patients is directly related to the risk of dissection-related events, including aneurysmal evolution, malperfusion and aortic rupture. In the present study, the long-term mortality was 4.0%, with a low rate of aortic-related death (0.5%), despite a high rate of aneurysmal evolution at 52.3%.These results are related to a close follow-up, reintervention in cases of complications and a low morbi-mortality rate associated with these reinterventions. Reinterventions occurred in most cases (82.1%) in the first 2 years after acute type A aortic dissection repair, Ohira et al. confirmed these results with the median duration from the index repair at 2 years [27]. 

Currently, endovascular therapy for the descending thoracic aorta remains the first-line therapy, but in cases of RAD, the primary entry tear is on the aortic arch in most cases, which is very challenging. Indeed, in our center and in most other aortic centers, hemiarch replacement is the most common repair for type A repair at the acute phase [27]. Sultan et al. proposed concomitant antegrade stent grafting of the descending thoracic aorta during transverse hemiarch reconstruction for acute type A aortic dissection repair, and they showed that TEVAR increased positive aortic remodeling on the descending thoracic aorta by promoting FL thrombosis (71.4% FL complete thrombosis on the descending thoracic aorta), with no increase in stroke, paraplegia or mortality rate [28]. A more aggressive repair at the acute phase with Frozen elephant trunk or open aortic arch repair prevent the risk of long-term evolution and increase the rate of FL thrombosis on the descending thoracic aorta at the cost of high mortality rate [4], so it would be beneficial in selected patients.

Open aortic arch surgery or frozen elephant trunk with prior ascending aortic replacement is challenging and is associated with a high rate of mortality, ranging from 5 to 20% [29,30,31]. In a recent study, among 117 reoperations after acute type A aortic dissection repair, Dell’Aquila et al. reported an in-hospital mortality of 19.6%. Furthermore, 31 patients underwent a distal reoperation (61.2% of total arch reoperation and 22.5% of FET), with an in-hospital mortality rate of 25.8% [32].

In our experience, hybrid treatment for RAD aneurysmal evolution could be a safe and effective technique with low rates of in-hospital mortality and morbidity. We reported the results of hybrid repair for chronic RAD [16] in a previous report, and among 28 elective patients, there was no perioperative death and one minor stroke. In 96.4% of cases, we observed complete FL thrombosis on the thoracic aorta. The present study confirms that hybrid repair for RAD is safe without death and only one minor stroke and is associated with 85.7% good anatomical results, including complete aortic remodeling and FL thrombosis on the thoracic aorta.

Branched endoprostheses could be a less invasive alternative to hybrid or open repair [33]. Maurel et al. reported 0% early mortality and 11% stroke after branched and fenestrated endoprostheses in aortic arch aneurysms, but it is well known that the rate of stroke after fenestrated or branched TEVAR is higher in aneurysms than in aortic dissections [34]. Indeed, in a recent study, the rate of in-hospital mortality and stroke was 4% in these specific patients with RAD [17]. Here, despite a low number of patients treated with branched endografts, we showed that this approach is safe without perioperative death or stroke. This technique will continue to be used and the indications will be extended to an increasing number of patients, especially for this indication.

Finally, we identified Marfan syndrome and bicuspid aortic valve as risk factors of distal reintervention, which confirms our previous results [21]. All epidemiological and anatomical risk factors identified here and, in the literature, could be used to increase the long-term survival of patients with a RAD. Indeed, we could offer them a more aggressive treatment at the acute or subacute phase to promote aortic remodeling and prevent aortic growth and late aortic rupture.

## 5. Conclusions

Despite a critical course in most cases of RAD, with a high rate of aneurysmal evolution and reintervention, the long-term mortality rate remains low with a close follow-up and a multidisciplinary management in an expert center.

These complex cases require a personalized therapeutic solution. Case-by-case discussion and management must remain a priority to increase the long-term survival of these patients.

## Figures and Tables

**Table 1:** Baseline characteristics.

Variables	Total n = 200
Age (years), mean (SD)	62.2 (11.6)
Male sex, n (%)	145 (72.5)
Hypertension, n (%)	121 (60.5)
Dyslipidemia, n (%)	40 (19.8)
Smoking, n (%)	36 (18.0)
Diabetes, n (%)	6 (3.0)
COPD, n (%)	11 (5.4)
Atrial fibrillation, n (%)	16 (7.8)
CAD, n (%)	16 (7.8)
Peripheral vascular disease, n (%)	7 (3.6)
Renal failure, n (%)	4 (1.8)
Marfan and related syndrome, n (%)	12 (6.0)
Bicuspid aortic, n (%)	8 (4.2)

SD, Standard deviation; CAD, Coronary artery disease; COPD, Chronic obstructive pulmonary disease.

**Table 2:** Baseline characteristics of patients with reintervention for distal aneurysmal evolution including aortic arch repair.

	Total n = 27 (100.0%)	Hybrid n = 21 (77.8%)	Branched Endograft n = 6 (22.2%)	*p* Value
Age (years), mean (SD)	63.0 +/−11.7	59.7 +/−10.7	74.7 +/−6.9	0.12
Male sex, n (%)	17 (63.0)	14 (66.7)	3 (50.0)	0.79
Hypertension, n (%)	15 (55.6)	11 (52.4)	4 (66.7)	0.87
Dyslipidemia, n (%)	2 (7.4)	1 (4.8)	1 (16.7)	0.92
Smoking, n (%)	4 (14.8)	2 (9.5)	2 (33.3)	0.42
Diabetes, n (%)	1 (3.7)	1 (4.8)	0 (0)	0.58
COPD, n (%)	4 (14.8)	3 (14.3)	1 (16.7)	0.77
Atrial fibrillation, n (%)	2 (7.4)	2 (9.5)	0 (0)	0.43
CAD, n (%)	1 (3.7)	1 (4.8)	0 (0)	0.58
Peripheral vascular disease, n (%)	1 (3.7)	1 (4.8)	0 (0)	0.58
Renal failure, n (%)	2 (7.4)	1 (4.8)	1 (16.7)	0.92
Marfan syndrome, n (%)	5 (18.5)	5 (23.8)	0 (0)	0.46

SD, Standard deviation; CAD, Coronary artery disease; COPD, Chronic obstructive pulmonary disease.

**Table 3:** Morbi-mortality after reintervention for distal aneurysmal evolution including aortic arch repair.

	Total n = 27 (100%)	Hybrid n = 21 (77.8%)	Branched Endograft n = 6 (22.2%)	*p* Value
**Hospital mortality**	0 (0)	0 (0)	0 (0)	NA
**Postoperative morbidity**	7 (25.9)	6 (28.6)	1 (16.7)	0.95
Stroke	1 (3.7)	1 (4.8)	0 (0)	0.58
Pulmonary complication	1 (3.7)	1 (4.8)	0 (0)	0.58
Carotid dissection	1 (3.7)	0 (0)	1 (16.7)	0.49
Major bleeding	3 (11.1)	3 (14.3)	0 (0)	0.80
Recurential paralysis	1 (3.7)	1 (4.8)	0 (0)	0.58
Medullary ischemia	0 (0)	0 (0)	0 (0)	NA
Number of hospitalizations	1.8 +/−0.7	1.8 +/−0.7	1.8 +/−1.0	0.96
Cumulative length of stay in intensive care unit	3.9 +/−2.0	4.4 +/−2.5	1.8 +/−0.8	<0.001
Cumulative length of stay in hospital	13.9 +/−5.3	14.8 +/−8.1	11.0 +/−4.2	0.14
**Anatomical results**				
Total aortic remodeling	11 (40.7)	10 (47.6)	1 (16.7)	0.37
FL thrombosis	12 (44.4)	8 (38.1)	4 (66.7)	0.44
FL partial thrombosis	4 (14.8)	3 (14.3)	1 (16.7)	0.88
Type Ia Endoleak	0 (0)	0 (0)	0 (0)	NA
Type Ib Endoleak	4 (14.8)	3 (14.3)	1(16.7)	0.88
Type II Endoleak	0 (0)	0 (0)	0 (0)	NA
Type III Endoleak	0 (0)	0 (0)	0 (0)	NA

## Data Availability

The data underlying this article will be shared upon reasonable request to the corresponding author.

## Abbreviations

CA	Circulatory Arrest
CAD	Coronary Artery Disease
CPB	Cardiopulmonary Bypass
COPD	Chronic Obstructive Pulmonary Disease
CT	Computed Tomography
IA	Innominate Artery
FL	False Lumen
LCCA	Left Common Carotid Artery
LSA	Left Subclavian Artery
RAD	Residual Aortic Dissection
TEVAR	Total Endo Vascular Aortic Repair

## References

[1] Gouveia E.M.R., Mourao M., Caldeira D., Alves M., Lopes A., Duarte A., Fernandes E.F.R., Mendes Pedro L. (2022). A systematic review and meta-analysis of the incidence of acute aortic dissections in population-based studies. J. Vasc. Surg..

[2] Evangelista A., Isselbacher E.M., Bossone E., Gleason T.G., Eusanio M.D., Sechtem U., Ehrlich M.P., Trimarchi S., Braverman A.C., Myrmel T. (2018). Insights From the International Registry of Acute Aortic Dissection: A 20-Year Experience of Collaborative Clinical Research. Circulation.

[3] Berretta P., Patel H.J., Gleason T.G., Sundt T.M., Myrmel T., Desai N., Korach A., Panza A., Bavaria J., Khoynezhad A. (2016). IRAD experience on surgical type A acute dissection patients: Results and predictors of mortality. Ann. Cardiothorac. Surg..

[4] Chabry Y., Porterie J., Gautier C.H., Nader J., Chaufour X., Alsac J.M., Reix T., Marcheix B., Koskas F., Ruggieri V.G. (2020). The frozen elephant trunk technique in an emergency: THORAFLEX French National Registry offers new insights. Eur. J. Cardio-Thorac. Surg. Off. J. Eur. Assoc. Cardio-Thorac. Surg..

[5] Isselbacher E.M., Preventza O., Black J.H., Augoustides J.G., Beck A.W., Bolen M.A., Braverman A.C., Bray B.E., Brown-Zimmerman M.M., Chen E.P. (2022). 2022 ACC/AHA Guideline for the Diagnosis and Management of Aortic Disease: A Report of the American Heart Association/American College of Cardiology Joint Committee on Clinical Practice Guidelines. J. Am. Coll. Cardiol..

[6] Kimura N., Itoh S., Yuri K., Adachi K., Matsumoto H., Yamaguchi A., Adachi H. (2015). Reoperation for enlargement of the distal aorta after initial surgery for acute type A aortic dissection. J. Thorac. Cardiovasc. Surg..

[7] Yeh C.H., Chen M.C., Wu Y.C., Wang Y.C., Chu J.J., Lin P.J. (2003). Risk factors for descending aortic aneurysm formation in medium-term follow-up of patients with type A aortic dissection. Chest.

[8] Inoue Y., Matsuda H., Omura A., Seike Y., Uehara K., Sasaki H., Kobayashi J. (2018). Long-term outcomes of total arch replacement with the non-frozen elephant trunk technique for Stanford Type A acute aortic dissection. Interact. Cardiovasc. Thorac. Surg..

[9] Suzuki T., Asai T., Kinoshita T. (2018). Predictors for Late Reoperation After Surgical Repair of Acute Type A Aortic Dissection. Ann. Thorac. Surg..

[10] Tamura K., Chikazawa G., Hiraoka A., Totsugawa T., Sakaguchi T., Yoshitaka H. (2017). The prognostic impact of distal anastomotic new entry after acute type I aortic dissection repair. Eur. J. Cardio-Thorac. Surg. Off. J. Eur. Assoc. Cardio-Thorac. Surg..

[11] Lederle F.A., Johnson G.R., Wilson S.E., Littooy F.N., Krupski W.C., Bandyk D., Acher C.W., Chute E.P., Hye R.J., Gordon I.L. (2000). Yield of repeated screening for abdominal aortic aneurysm after a 4-year interval. Aneurysm Detection and Management Veterans Affairs Cooperative Study Investigators. Arch. Intern. Med..

[12] Conzelmann L.O., Hoffmann I., Blettner M., Kallenbach K., Karck M., Dapunt O., Borger M.A., Weigang E., Investigators G. (2012). Analysis of risk factors for neurological dysfunction in patients with acute aortic dissection type A: Data from the German Registry for Acute Aortic Dissection type A (GERAADA). Eur. J. Cardio-Thorac. Surg. Off. J. Eur. Assoc. Cardio-Thorac. Surg..

[13] Tasoudis P.T., Magouliotis D.E., Varvoglis D.N., Ziogas I.A., Salmasi M.Y., Spanos K., Kourliouros A., Matsagkas M., Giannoukas A., Athanasiou T. (2022). Proximal versus extensive repair in acute type A aortic dissection: An updated systematic review and meta-analysis. Gen. Thorac. Cardiovasc. Surg..

[14] Berger T., Kreibich M., Mueller F., Rylski B., Kondov S., Schrofel H., Pingpoh C., Beyersdorf F., Siepe M., Czerny M. (2021). The frozen elephant trunk technique for aortic dissection is safe after previous aortic repair. Eur. J. Cardio-Thorac. Surg. Off. J. Eur. Assoc. Cardio-Thorac. Surg..

[15] Demal T.J., Bax L., Brickwedel J., Kolbel T., Vettorazzi E., Sitzmann F., Reichenspurner H., Detter C. (2021). Outcome of the frozen elephant trunk procedure as a redo operation. Interact. CardioVascular Thorac. Surg..

[16] Gaudry M., Porto A., Blanchard A., Chazot J.V., Bal L., De Masi M., Bartoli A., Barral P.A., Jacquier A., Gariboldi V. (2022). A 10-Year Aortic Center Experience with Hybrid Repair of Chronic “Residual” Aortic Dissection After Type A Repair. Cardiovasc. Drugs Ther..

[17] Verscheure D., Haulon S., Tsilimparis N., Resch T., Wanhainen A., Mani K., Dias N., Sobocinski J., Eagleton M., Ferreira M. (2019). Endovascular Treatment of Post Type A Chronic Aortic Arch Dissection With a Branched Endograft: Early Results From a Retrospective International Multicenter Study. Ann. Surg..

[18] Nana P., Spanos K., Dakis K., Giannoukas A., Kölbel T., Haulon S. (2022). Systematic Review on Customized and Non-customized Device Techniques for the Endovascular Repair of the Aortic Arch. J. Endovasc. Ther. Off. J. Int. Soc. Endovasc. Spec..

[19] Kimura N., Tanaka M., Kawahito K., Yamaguchi A., Ino T., Adachi H. (2008). Influence of patent false lumen on long-term outcome after surgery for acute type A aortic dissection. J. Thorac. Cardiovasc. Surg..

[20] Gaudry M., Guivier-Curien C., Blanchard A., Porto A., Bal L., Omnes V., De Masi M., Lu C., Jacquier A., Piquet P. (2022). Volume Analysis to Predict the Long-Term Evolution of Residual Aortic Dissection after Type A Repair. J. Cardiovasc. Dev. Dis..

[21] Gaudry M., Porto A., Guivier-Curien C., Blanchard A., Bal L., Resseguier N., Omnes V., De Masi M., Ejargue M., Jacquier A. (2022). Results of a prospective follow-up study after type A aortic dissection repair: A high rate of distal aneurysmal evolution and reinterventions. Eur. J. Cardio-Thorac. Surg. Off. J. Eur. Assoc. Cardio-Thorac. Surg..

[22] Faure E.M., El Batti S., Abou Rjeili M., Julia P., Alsac J.M. (2018). Mid-term Outcomes of Stent Assisted Balloon Induced Intimal Disruption and Relamination in Aortic Dissection Repair (STABILISE) in Acute Type B Aortic Dissection. Eur. J. Vasc. Endovasc. Surg..

[23] Faure E.M., El Batti S., Sutter W., Bel A., Julia P., Achouh P., Alsac J.M. (2019). Stent-assisted balloon-induced intimal disruption and relamination of distal remaining aortic dissection after acute DeBakey type I repair. J. Thorac. Cardiovasc. Surg..

[24] Vecchini E., Gulmini M., Peluso A., Fasoli G., Anselmi A., Maluta T., De Cristan D., Magnan B., Ricci M. (2022). The treatment of irreparable massive rotator cuff tears with inspace balloon: Rational and medium-term results. Acta Bio Med. Atenei Parm..

[25] Rohlffs F., Tsilimparis N., Panuccio G., Heidemann F., Behrendt C.A., Kölbel T. (2022). The Knickerbocker Technique: Technical Aspects and Single-Center Results of a New Endovascular Method for False Lumen Occlusion in Chronic Aortic Dissection. J. Endovasc. Ther. Off. J. Int. Soc. Endovasc. Spec..

[26] Fann J.I., Smith J.A., Miller D.C., Mitchell R.S., Moore K.A., Grunkemeier G., Stinson E.B., Oyer P.E., Reitz B.A., Shumway N.E. (1995). Surgical management of aortic dissection during a 30-year period. Circulation.

[27] Ohira S., Malekan R., Kai M., Goldberg J.B., Laskowski I., De La Pena C., Mason I., Lansman S.L., Spielvogel D. (2022). Aortic Reoperation After Prior Acute Type A Aortic Dissection Repair: Don’t Despair the Repair. Ann. Thorac. Surg..

[28] Sultan I., Wallen T.J., Habertheuer A., Siki M., Arnaoutakis G.J., Bavaria J., Szeto W.Y., Milewski R., Vallabhajosyula P. (2017). Concomitant antegrade stent grafting of the descending thoracic aorta during transverse hemiarch reconstruction for acute DeBakey I aortic dissection repair improves aortic remodeling. J. Card. Surg..

[29] Quintana E., Bajona P., Schaff H.V., Dearani J.A., Daly R.C., Greason K.L., Pochettino A. (2014). Open aortic arch reconstruction after previous cardiac surgery: Outcomes of 168 consecutive operations. J. Thorac. Cardiovasc. Surg..

[30] Malvindi P.G., van Putte B.P., Sonker U., Heijmen R.H., Schepens M.A., Morshuis W.J. (2013). Reoperation after acute type a aortic dissection repair: A series of 104 patients. Ann. Thorac. Surg..

[31] Di Bartolomeo R., Berretta P., Pantaleo A., Murana G., Cefarelli M., Alfonsi J., Barberio G., Leone A., Di Marco L., Pacini D. (2017). Long-Term Outcomes of Open Arch Repair After a Prior Aortic Operation: Our Experience in 154 Patients. Ann. Thorac. Surg..

[32] Dell’Aquila A.M., Pollari F., Fattouch K., Santarpino G., Hillebrand J., Schneider S., Landwerht J., Nasso G., Gregorini R., Del Giglio M. (2017). Early outcomes in re-do operation after acute type A aortic dissection: Results from the multicenter REAAD database. Heart Vessel..

[33] Haulon S., Greenberg R.K., Spear R., Eagleton M., Abraham C., Lioupis C., Verhoeven E., Ivancev K., Kolbel T., Stanley B. (2014). Global experience with an inner branched arch endograft. J. Thorac. Cardiovasc. Surg..

[34] Maurel B., Mastracci T.M., Spear R., Hertault A., Azzaoui R., Sobocinski J., Haulon S. (2016). Branched and fenestrated options to treat aortic arch aneurysms. J. Cardiovasc. Surg..

